# The Patient and Treatment Characteristics of Kidney Transplant Recipients with a Clinically Relevant Jaffe/Enzymatic Serum Creatinine Difference

**DOI:** 10.3390/jcm14051668

**Published:** 2025-02-28

**Authors:** Kristina Boss, Susanne Stolpe, André Müller, Justa Friebus-Kardash, Bernd Wagner, Marc Wichert, Roland Assert, Lothar Volbracht, Andreas Stang, Bernd Kowall, Andreas Kribben

**Affiliations:** 1 Department of Nephrology, University Hospital Essen, University Duisburg-Essen, 45147 Essen, Germany; 2 Institute of Medical Informatics, Biometry and Epidemiology, University Hospital Essen, University Duisburg-Essen, 45147 Essen, Germany; 3 Department of Clinical Chemistry, University Hospital Essen, University Duisburg-Essen, 45147 Essen, Germany

**Keywords:** acute kidney injury, creatinine, enzymatic method, Jaffe method, kidney transplantation, immunosuppression

## Abstract

**Background:** Differences in serum creatinine (SCr) between the Jaffe and enzymatic methods affect the detection and staging of chronic kidney disease in kidney transplant recipients (KTRs). However, there are very limited data on the extent to which the detection of acute kidney injury (AKI) is affected, what impact immunosuppression can have and whether a KTR-specific estimated glomerular filtration rate (eGFR) formula is beneficial. **Methods:** A total of 12,081 parallel Jaffe/enzymatic SCr (eSCr) measurements of adult outpatient KTRs (61% male, median age 53 years) in the same serum sample at the University Hospital Essen (Germany) between January 2020 and October 2023 were evaluated. AKI and CKD were defined according to current KDIGO guidelines. The GFR was estimated using CKD-EPI and KTR-specific formulas. **Results:** In about 1% of all measurements and 5% of the KTR patients, the SCr difference between the two methods was ≥ 0.3 mg/dl. A total of 81% of these patients were male; the median age was 52 years. High levels of immunosuppression, including when Belatacept was used, did not seem to have a clinically relevant impact on the difference between Jaffe and eSCr. The KTR-specific eGFR formula generally showed a greater agreement between Jaffe and eSCr than the CKD-EPI eGFR formula, but they showed differences in the classification of CKD stages, especially in less severe stages. **Conclusions:** Clinically relevant SCr differences between Jaffe and SCr are rare and depend on the type of immunosuppression. A KTR-specific eGFR formula could be beneficial in some cases, but there are limitations in less severe CKD stages.

## 1. Introduction

The determination of renal function is essential in the treatment of patients with acute kidney injury (AKI) and chronic kidney disease (CKD). The measurement of serum creatinine (SCr) and the estimation of the glomerular filtration rate (eGFR) derived from this are the main criterions. SCr can be measured by different techniques. The Jaffe method and the enzymatic method are the most frequently used methods, despite the gold standard isotope dilution mass spectrometry [1,2]. Differences between Jaffe SCr and enzymatic SCr (eSCr) have an effect on both kidney disease detection and staging. In a large cohort of outpatients and inpatients of several medical disciplines, around one in five parallel SCr determinations led to deviating CKD stages; in outpatient kidney transplant recipients (KTRs), this occurred in about one in seven parallel SCr determinations [3,4]. SCr differences between the Jaffe and enzymatic methods are of particular importance in KTRs as they can influence the management of several clinically relevant scenarios.

First, in rare cases, the difference between Jaffe and eSCr can be ≥ 0.3 mg/dl, so the definition of AKI is fulfilled [3,5]. Such large SCr deviations would be even more relevant for KTRs, as they are always suspicious of an acute kidney rejection. At present, there are no data available on the frequency with which such an SCr difference could result in the misdiagnosis of AKI in outpatient KTR. The difference between Jaffe and eSCr could also be due to interference with immunosuppression. It is known that immunosuppressive drugs, like tacrolimus and mycophenolate mofetil (MMF), influence SCr clearance and the GFR [6]. Very high tacrolimus levels are nephrotoxic and can lead to AKI [7]. It is not yet clear whether this also plays a role in deviations between Jaffe and eSCr.

Second, there are more and more KTRs whose immunosuppressive therapy includes Belatacept. This fusion protein blocks a T-cell activation pathway and is administered in fixed doses without measuring serum levels [8,9]. There are only very limited data on to what extent Belatacept affects the difference in SCr between Jaffe and enzymatic SCr [10]. A method for drug monitoring has only recently been developed [11].

Third, little is known about the characteristics of patients for whom a different CKD stage is estimated as a consequence of discrepancies between Jaffe and eSCr. A higher-grade CKD stage, in particular the transition between stages G2 and G3a, is of high importance for outpatient care, as this is where the boundary between screening and treatment is drawn, according to the current Kidney Disease Improving Global Outcomes (KIDGO) guidelines [1]. Control intervals should be shortened, and further diagnostics should be initiated at this stage. It is also important to note that a change from CKD stage G2 to G3a is relevant even in cases where KTRs are already undergoing regular outpatient care because it affects, for example, the frequency of check-ups at the transplant centre and the need for dose adjustments to many medications. In this context, it is also unclear whether the application of the kidney recipient-specific GFR (KRS-eGFR) equation developed by Raynaud et al. [12] leads to fewer changes in the CKD stage.

The aim of this study was therefore to investigate to what extent an SCr difference between the Jaffe and enzymatic methods could lead to the misdiagnosis of AKI in outpatient KTRs and whether immunosuppressive drugs influence these differences. A further aim of this study was to investigate the patient and treatment characteristic of KTRs with a change in CKD stage due to the serum creatinine measurement method and the potential benefits of the KRS-eGFR equation in such cases.

## 2. Materials and Methods

### 2.1. Study Design

A retrospective analysis was conducted on all consecutive measurements made between January 2020 and October 2023 using the Jaffe method and the eSCr method in the corresponding serum samples of adult outpatient kidney transplant recipients of the Department of Nephrology at University Hospital Essen, Essen, Germany. The two SCr measurements were carried out simultaneously. In all measurements, both Jaffe and enzymatic SCr levels were determined due to internal standards for elective and non-elective visits. Patients were included in this study regardless of the reason for their visit to the renal transplant outpatient clinic. Measurements were analysed for both elective and non-elective visits. The final number of patients included in this study was 12,081, 61% of whom were male.

Tacrolimus levels were determined by one-step immunoassay; MMF levels were determined by an enzyme multiplied immunoassay technique (Abbott Laboratories, Abbott Park, IL, USA). We used a chemiluminescent immunoassay also from Abbott Laboratories for the measurement of ciclosporin serum levels and Liquid chromatography–mass spectrometry for the determination of everolimus serum levels. The Department of Clinical Chemistry of the University Hospital Essen conducted all analyses. To determine Jaffe SCr, we used Atellica measurement systems (Atellica 930 analyser, Atellica CH Crea_2 assay, Siemens Healthcare Diagnostics, Marburg, Germany) for the correlation of patient samples to the reference material SRM967 from the National Institute of Standards and Technology (NIST). During the entire study period, we used the same IDMS traceable method. The same applied to the determination of eSCr. Further details of the SCr measurement methods and the study cohort are described elsewhere [3,4]. Belatacept was dosed according to professional information and applied intravenously every four weeks as a maintenance therapy.

Chronic kidney disease and acute kidney injury were defined according to current KDIGO guidelines [1,5]. The eGFR was utilized as a marker of renal function. It was calculated using the CKD-EPI 2009 formula for SCr and the kidney recipient-specific GFR (KRS-eGFR) equation developed by Raynaud et al. [12,13]. The CKD-EPI formula was used without adjusting for race as recommended for use in Europe [14,15]. Also, in the study population, more than 99% of patients are Caucasians. We also decided to investigate the KRS-eGFR formula, as this may provide more accuracy in the specific patient population presented here.

Applying the CKD-EPI eGFR formula, two groups were defined, in which a difference between Jaffe and eSCr led to different stagings of CKD in the context of particular sensitivity with regard to CKD progression: group 1 included patients with CKD stage G2 according to eSCr but stage G3a according to Jaffe SCr, and group 2 included patients with CKD stage G3a according to eSCr but stage G2 according to Jaffe SCr.

### 2.2. Statistical Analysis

To characterize the study population, analyses of frequency distributions, central tendency and variability measurements were conducted. In the majority of patients, a double measurement (Jaffe and SCr) was performed more than once. All double measurements were used statistically for this study. The agreement between the stages of CKD classification was assessed using kappa values [16]. SAS (version 9.4; Cary, NC, USA) and GraphPad Prism (version 10.3.1; San Diego, CA, USA) software programs were used for all statistical analyses and graphical evaluations.

### 2.3. Ethics Approval

This study was performed in accordance with the Declaration of Helsinki and the International Conference on Harmonization Good Clinical Practice guidelines. This study was approved by the local ethics committee of the University of Duisburg-Essen (20-9501-BO).

## 3. Results

### 3.1. Study Population Characteristics

A total of 12,081 parallel serum creatinine measurements were evaluated for 1246 outpatient kidney transplant recipients. Of the patients, 61% (n = 7370) were male. The mean age of the participants was 50 years (range 18–90 years, median 53 years). The characteristics of the study population are outlined in [3].

### 3.2. The Impact of the Measurement Method on the Diagnosis of AKI

There were 99 measurements (0.8%) of 69 patients with an SCr difference between the Jaffe and enzymatic methods ≥ 0.3 mg/dl. A total of 81% of these patients were male; the median age was 52 years (range 18–76 years). The average differences between Jaffe and eSCr in these 99 measurements were 0.43 mg/dl (maximum difference 1.61 mg/dl) and 4.8 mL/min/1.73 m^2^ eGFR (Table 1). Even though such a large SCr difference was rare overall, it was noticeably more frequent in patients between the ages of 18 and 29, which was one quarter of all measurements with an SCr difference ≥ 0.3 mg/dl (1.7% of the total cohort). In 75 out of 99 measurements, eSCr was higher than Jaffe SCr; in the other 24 measurements, Jaffe SCr was higher than eSCr. In 17 of these 69 patients, there was an SCr difference between the Jaffe and enzymatic methods ≥ 0.3 mg/dl in more than one measurement (range: 2–5 measurements). These patients were all male; the median age was 39 years (range: 18–64 years).

Measurements included all common combinations of immunosuppressive therapy (Supplementary Table S1). Proportionally, a clinically relevant SCr difference was found most frequently in patients with a dual immunosuppression consisting of ciclosporin/prednisone (2% of all measurements). Only rarely was such a discrepancy found in patients with an immunosuppressive therapy consisting of tacrolimus/prednisone (1% of all measurements). In patients with the most common therapy combination of tacrolimus, MMF and prednisone, such large deviations between the SCr measurement methods were also found in 1% of all measurements.

We identified 5120 SCr measurements from female patients with an immunosuppressive therapy consisting of tacrolimus. The median tacrolimus level was 6.1 ng/mL (IQR 5.0–7.5 ng/mL; range 0.8–42.2 ng/mL). The median Jaffe/eSCr deviation here was 0.06 mg/dl (IQR 0.03–0.10 mg/dl). In this group, there was only one measurement with an SCr difference between Jaffe and eSCr ≥ 0.3 mg/dl. Among measurements with high tacrolimus levels > 10.0 ng/mL (n = 291 measurements), none of these measurements showed such a large deviation that an AKI misdiagnosis could occur. The median SCr difference in this subgroup was 0.06 mg/dl.

We identified 6611 SCr measurements from male patients with an immunosuppressive therapy consisting of tacrolimus. The median tacrolimus level was 6.3 ng/mL (IQR 5.1–7.8 ng/mL; range 0.8–60.0 ng/mL). The median Jaffe/eSCr deviation here was 0.07 mg/dl (IQR 0.04–0.11 mg/dl). In this group, there were 85 measurements with an SCr difference between Jaffe and eSCr ≥ 0.3 mg/dL. A total of 12 of these measurements were detected in samples with high tacrolimus levels > 10.0 ng/mL. In total, there were 508 samples with such a high tacrolimus level. The median SCr difference in this subgroup was also 0.06 mg/dL.

### 3.3. The Impact of Belatacept on the Difference Between Jaffe and Enzymatic SCr Measurements

In 25 patients (11 women and 14 men), Belatacept was part of the immunosuppressive therapy. The median age here was 58 years (range 18–82 years). A total of 800 parallel SCr measurements were available for evaluation. The median frequency was 33 measurements per patient (IQR 23–38 measurements/patient). Belatacept was mostly combined with tacrolimus, in some cases also with everolimus (Supplementary Table S1). The average difference between the Jaffe and eSCr methods in these patients was 0.01 mg/dl. A total of 95% of the differences in SCr between the two measurement methods were between −0.17 mg/dl and 0.20 mg/dl. The corresponding average eGFR difference was −1.3 mL/min/1.72 m^2^ (LoA 5.3 mL/min/1.72 m^2^/−8.0 mL/min/1.72 m^2^). Only 0.5% of all measurements with a Belatacept-based immunosuppressive therapy showed an SCr difference ≥ 0.3 mg/dl (five measurements: four from male patients, one from a female patient).

### 3.4. Patient Characteristics and the Impact of the Kidney Recipient-Specific GFR (KRS-eGFR) Equation in Cases of Deviating CKD Stages Due to the Jaffe/eSCr Difference

We identified 543 measurements of 265 patients with CKD stage G2 according to eSCr but stage G3a according to Jaffe SCr (group 1) and 104 measurements of 67 patients with CKD stage G3a according to eSCr but stage G2 according to Jaffe SCr (group 2). A total of 37 of these 332 patients (76% male, median age 40 years, range 18–70 years) had measurements that were sometimes assigned to group 1 and sometimes to group 2. There were 462 measurements from patients with an immunosuppressive therapy consisting of tacrolimus, MMF and prednisone; 14 measurements under the influence of tacrolimus, Belatacept and prednisone; 34 measurements with tacrolimus, everolimus and prednisone; and 15 measurements with ciclosporin, MMF and prednisone. Also, there were 18 measurements of patients with a dual immunosuppression with tacrolimus and prednisone. The median serum level was 6.2 ng/mL (IQR 5.1–7.7 ng/mL) for tacrolimus and 111 ng/mL (IQR 78–193 ng/mL) for ciclosporin. The median everolimus level was 4.2 ng/mL (IQR 3.6–5.0 ng/mL). Detailed patient characteristics of both groups are presented in Table 2. The two groups differ from each other in terms of demographics and comorbidities, most likely due to the different sample sizes. Although most CKD stage deviations are observed between the stages G2 and G3a, there do not seem to be any clear characteristics of these patients based on the parameters recorded here.

Using Jaffe and eSCr in conjunction with the KRS eGFR formula, 1761 (14.6%) of the measurements yielded a deviating CKD classification (Table 3). The kappa value here was 0.805 (95% CI 0.80–0.81). A comparison of the CKD-EPI and KRS eGFR formulas reveals that the KRS formula exhibits a greater number of deviations in the less severe CKD stages than the CKD-EPI formula (Table 4).

## 4. Discussion

### 4.1. Key Findings

This study evaluated the impact of the serum creatinine difference between the Jaffe and enzymatic methods in clinically relevant settings like suspected AKI/acute rejection and the deviation of CKD stages in outpatient KTRs. We found that in approximately 1% of all SCr determinations in these patients, the SCr difference between the Jaffe and enzymatic methods is so large that the misdiagnosis of AKI is possible. This affected young men, regardless of immunosuppressive therapy. Belatacept did not seem to have a relevant impact on the difference between Jaffe and eSCr, as both the median difference between these two measurement methods was smaller and the cases of possible AKI misdiagnosis were less frequent under this type of immunosuppression. Compared to the CKD-EPI eGFR formula, the KRS eGFR formula showed a slightly higher agreement of the CKD stages depending on the SCr method, using kappa values. However, the KRS eGFR formula resulted in more deviations in the less severe CKD stages than the CKD-EPI eGFR formula.

### 4.2. Comparison with Previous Studies and Prospects

The difference between the Jaffe and enzymatic SCr measurement methods has been the subject of investigation in several studies conducted in a variety of clinical settings [17,18,19,20]. In previous studies, we showed that this SCr difference has a pronounced impact on everyday clinical practice, influencing both the diagnosis and staging of acute and chronic kidney disease [3,4]. These findings were in line with previous studies by Drion et al. and Gottlieb et al. [21,22]. Furthermore, this allowed us to demonstrate that a substantial sample size is essential for evaluating the potential impact of the slight discrepancy between the two SCr measurement methods, given the relatively small absolute difference between the two. The same applied to kidney transplant recipients, a subgroup in which SCr differences and thus also eGFR differences and deviating CKD stages are of particular relevance. A special feature of this subgroup is the possible influence of immunosuppressants on the SCr difference. It is known that tacrolimus, one of the most used immunosuppressive drugs, can reduce the GFR and renal tubular creatinine secretion by inhibiting cyclooxygenase 2 in the renal medulla [6]. Further, donor–recipient mismatch and the presence of a single kidney metabolism have been considered when assessing kidney function in these patients [23,24]. In their recent study, Weidmann et al. investigated such aspects in a KTR cohort, especially the potential benefits of using cystatin C [10]. They pointed out that there are several clinical scenarios like the use of Belatacept or prednisone that are associated with smaller or greater differences between eGFRcr and eGFRcys equations. Regarding the impact of Belatacept, our study confirms their finding. We also found a trend in that Belatacept does not seem to affect SCr or eGFR differences. Large SCr differences in samples from patients with a Belatacept-based therapy were less frequent than with other forms of immunosuppressive therapy. Also, the median SCr and eGFR difference was lower in these samples than in the total cohort. Nevertheless, the number of measurements in the Belatacept subgroup was not so large, as Belatacept is a rather rarely used immunosuppressant. Therefore, only limited conclusions can be drawn from these data. So, we can thus supplement the previous findings on the influence of immunosuppression on SCr and cystatin C with impacts on different SCr measurement methods.

Previous studies have demonstrated that eGFR equations exhibit variability in their performance when compared to the measured GFR (mGFR) in KTRs [25,26,27,28]. Considering the aforementioned shortcomings, a recently published, race-free, kidney recipient-specific eGFR equation, tailored to the needs of KTRs, was developed with the aim of overcoming these inconsistencies [12]. Its performance was comparable to that of existing SCr-based eGFR equations, such as the CKD-EPI 2009 and 2021 equations [29,30]. Accordingly, we could show in our study that the agreement in terms of kappa values between Jaffe and enzymatic SCr was higher when the KRS eGFR formula was applied compared to when the CKD-EPI or EKFC eGFR formula was applied. Nevertheless, there are discrepancies between the various eGFR formulas with regard to the consistency of SCr methods, which vary according to the CKD stages. Nevertheless, the KRS eGFR formula yielded a greater number of discrepancies in the less severe CKD stages than the CKD-EPI or EKFC eGFR formula.

## 5. Strengths and Limitations

Our study has several strengths and limitations. To our knowledge, this is the first study that evaluated the patient and treatment characteristics of KTRs with a clinically relevant serum creatinine difference due to the measurement methods used (Jaffe or enzymatic method). One of the strengths of this study is the large cohort, which also made it possible to analyse the influence of less frequently used immunosuppressive drugs, such as Belatacept. The present study also offers preliminary insights into the patients’ characteristics in whom an SCr deviation due to the measurement method used may have a pronounced impact on clinical assessment, potential further diagnosis and subsequent treatment.

It is important to note, however, that there are limitations that must be considered. The absence of data pertaining to cystatin C precluded the application of corresponding eGFR formulas. This is particularly relevant in patients with CKD at stage 2 or 3a. Additionally, the CKD-EPI eGFR equation was applied using Jaffe SCr. It should be noted that the eGFR equation was only validated using eSCr. However, it is highly probable that Jaffe SCr measurements are used to estimate the GFR in clinical contexts. A further limitation is the absence of a measured GFR or the use of a gold standard technique.

Moreover, this study did not examine the potential impact of haemolysis, icterus and lipemia (HIL) indices. It is possible that both Jaffe SCr and eSCr may increase due to the presence of interfering substances, such as glucose, bilirubin or pharmaceutical agents. The serum levels of these parameters were not assessed in the present study. Previous analyses of HIL indices demonstrated markedly elevated concentrations of, for instance, conjugated bilirubin in less than 1% of measurements [3]. However, it is plausible that individuals with diabetes and elevated serum glucose levels may exhibit a distinct degree of discrepancy between Jaffe and enzymatic serum creatinine compared to non-diabetic subjects.

## 6. Conclusions

The present study showed that SCr differences between the Jaffe and enzymatic methods ≥ 0.3 mg/dl are rare overall, but they affect rather young men, regardless of the type of immunosuppressive therapy. The use of the KTR-specific eGFR formula showed a generally greater agreement between Jaffe and eSCr than with the CKD-EPI and EKFC eGFR formulas, but there were differences in the classification of CKD stages, especially in less severe stages.

## Figures and Tables

**Table 1:** The proportion of measurements with a difference in serum creatinine between the Jaffe and enzymatic methods ≥0.3 mg/dL in adult outpatient kidney transplant recipients at the University Hospital of Essen, Essen, Germany, 2020–2023.

Characteristic	N All	N SCr Difference ≥ 0.3 mg/dL	[%] 95% CI
**Sex**			
**All**	12,081	99	0.8 (0.67–1.00)
Male	7370	85	1.2 (0.92–1.42)
Female	4711	14	0.3 (0.18–0.50)
**Age (years)**			
18–29	1434	25	1.7 (1.20–2.60)
30–39	2159	12	0.6 (0.32–0.97)
40–49	1545	18	1.2 (0.74–1.8)
50–59	2888	19	0.7 (0.42–1.00)
60–69	2809	16	0.6 (0.35–0.92)
70–79	1134	9	0.8 (0.42–1.5)
≥ 80	112	0	-

Abbreviations: SCr: serum creatinine. N reflects the number of measurements.

**Table 2:** The characteristics of adult outpatient kidney transplant recipients with deviating CKD stages between stages G2 and G3a due to a serum creatinine difference between the Jaffe and enzymatic methods at University Hospital Essen, Essen, Germany, 2020–2023.

Characteristics	Group 1 265 Patients	Group 2 67 Patients
Age [years], median (range)	53 (18–83)	40 (18–70)
Male [n%]	55.8	70.1
BMI (median, IQR)	25.1 (22.1–27.9)	25.6 (22.2–27.9)
Renal disease [n%]		
Diabetic NP	11.3	6.7
Hypertensive NP	15.1	10.0
GN (IgA)	15.5 (14.3)	13.3 (3.4)
ADPKD	12.8	16.7
Malformations	14.3	26.7
Rare causes	9.7	13.3
Unknown	6.8	9.9
Hypertension [n%]	93.6	96.0
Diabetes mellitus [n%]	24.5	10.0
Time since Tx [month]	35 (17–66)	138 (31–215)
eSCr median (IQR)	1.18 (1.01–1.29)	1.35 (1.22–1.52)
Jaffe SCr median (IQR)	1.29 (1.12–1.40)	1.28 (1.14–1.42)
eGFR_eSCr_ median (IQR)	57.5 (55.7–58.9)	61.2 (60.4–62.3)
eGFR_Jaffe-SCr_ median (IQR)	62.7 (61.1–64.8)	58.3 (57.1–59.1)

Abbreviations: body mass index: BMI; nephropathy: NP; glomerulonephritis: GN; autosomal dominant polycystic kidney disease: ADPKD; rare causes including thrombotic microangiopathy: TMA; systemic lupus erythematode: SLE; transplantation: Tx; enzymatic serum creatinine: eSCr; serum creatinine: SCr [mg/dL]; estimated glomerular filtration rate: eGFR (mL/min/1.73 m^2^); interquartile range: IQR. Each patient was analysed only once, even if they received multiple parallel SCr measurements. eGFR was determined by applying CKD-EPI formula. In group 1 (G2_eSCr_/G3a_Jaffe-SCr_), 543 measurements were analysed, in group 2 (G3a_eSCr_/G2_Jaffe-SCr_), 104 measurements.

**Table 3:** CKD stages depending on SCr measurement method (KRS eGFR formula) of adult outpatient kidney transplant recipients at University Hospital Essen, Essen, Germany, 2020–2023.

ENZYM	G1	G2	G3A	G3B	G4	G5	TOTAL
JAFFE							
**G1**	142	129	0	0	0	0	271
**G2**	102	2275	92	0	0	0	2469
**G3A**	0	886	3310	87	1	0	4284
**G3B**	0	3	278	2964	77	0	3322
**G4**	0	0	0	96	1451	4	1551
**G5**	0	0	0	0	6	178	184
**TOTAL**	244	3293	3680	3147	1535	182	**12,081**

Abbreviations: SCr: serum creatinine; eGFR: estimated glomerular filtration rate. eGFR according to KRS formula [12].

**Table 4:** Deviation of CKD stages depending on eGFR formula in outpatient kidney transplant recipients at University Hospital Essen, Essen, Germany, 2020–2023.

	CKD-EPI	KRS
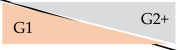	32.7%	41.8%
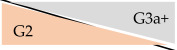	16.9%	27.0%
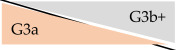	11.3%	7.6%
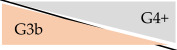	4.8%	3.1%
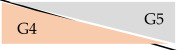	0.8%	0.3%

Abbreviations: eGFR: estimated glomerular filtration rate. eGFR according to CKD-EPI formula 2009 [13] and KRS formula [12]. Orange background refers to CKD stage with enzymatic serum creatinine; grey background corresponds to CKD stage with Jaffe serum creatinine. Percentage numbers show proportion of deviated CKD classification (e.g., according to enzymatic SCr, 32.7% of all measurements with CKD stage G1 had CKD stage G2 according to corresponding Jaffe SCr). Percentage numbers of upgrading effects are not shown.

## Data Availability

The data underlying this article will be shared on reasonable request to the corresponding author.

## Supplementary Materials

The following supporting information can be downloaded at https://www.mdpi.com/article/10.3390/jcm14051668/s1, Table S1: Proportion of immunosuppressive therapy of adult outpatient kidney transplant recipients at University Hospital Essen, Essen, Germany, 2020–2023.

## Abbreviations

AKI	acute kidney injury
CKD	chronic kidney disease
EKFC	European Kidney Function Consortium
eGFR	estimated glomerular filtration rate
eSCr	enzymatic serum creatinine
HIL	haemolysis, icterus and lipemia
KDIGO	Kidney Disease Improving Global Outcomes
KRS	kidney recipient-specific
KTR	kidney transplant recipient
MMF	mycophenolate mofetil
SCr	serum creatinine

## References

[1] Kidney Disease: Improving Global Outcomes (KDIGO) CKD Work Group (2024). KDIGO 2024 Clinical Practice Guideline for the Evaluation and Management of Chronic Kidney Disease. Kidney Int..

[2] Brück K., Jager K.J., Dounousi E., Kainz A., Nitsch D., Ärnlöv J., Rothenbacher D., Browne G., Capuano V., Ferraro P.M. (2015). Methodology used in studies reporting chronic kidney disease prevalence: A systematic literature review. Nephrol. Dial. Transplant..

[3] Boss K., Stolpe S., Müller A., Wagner B., Wichert M., Assert R., Volbracht L., Stang A., Kowall B., Kribben A. (2023). Effect of serum creatinine difference between the Jaffe and the enzymatic method on kidney disease detection and staging. Clin. Kidney J..

[4] Boss K., Stolpe S., Müller A., Friebus-Kardash J., Wagner B., Wichert M., Assert R., Volbracht L., Stang A., Kowall B. (2024). Effect of Difference in Serum Creatinine between Jaffe and Enzymatic Methods in Outpatient Kidney Transplant Recipients. J. Clin. Med..

[5] Kellum J.A., Lameire N., Aspelin P., Barsoum R.S., Burdmann E.A., Goldstein S.L., Herzog C.A., Joannidis M., Kribben A., Levey A.S. (2012). Kidney Disease: Improving Global Outcomes (KDIGO) Acute Kidney Injury Work Group Clinical Practice Guideline for Acute Kidney Injury. Kidney Int. Suppl..

[6] Jacobson H.R. (1979). Altered permeability in the proximal tubule response to cyclic AMP. Am. J. Physiol..

[7] Bentata Y. (2020). Tacrolimus: 20 years of use in adult kidney transplantation. What we should know about its nephrotoxicity. Artif. Organs..

[8] Martin S.T., Tichy E.M., Gabardi S. (2011). Belatacept: A Novel Biologic for Maintenance Immunosuppression After Renal Transplantation. Pharmacother. J. Hum. Pharmacol. Drug Ther..

[9] Vincenti F., Rostaing L., Grinyo J., Rice K., Steinberg S., Gaite L., Moal M.-C., Mondragon-Ramirez G.A., Kothari J., Polinsky M.S. (2016). Belatacept and Long-Term Outcomes in Kidney Transplantation. N. Engl. J. Med..

[10] Weidmann L., Laux C., Castrezana Lopez K., Harmacek D., George B., von Moos S., Schachtner T. (2024). Immunosuppression and transplantation-related characteristics affect the difference between eGFR equations based on creatinine compared to cystatin C in kidney transplant recipients. Clin. Kidney J..

[11] Chhun S., Trauchessec M., Melicine S., Nicolas F., Miele A., Lukic S., Vilain E., Amrouche L., Lebert D., Anglicheau D. (2023). A Validated LC-MS/MS Method for Performing Belatacept Drug Monitoring in Renal Transplantation. Biomedicines.

[12] Raynaud M., Al-Awadhi S., Juric I., Divard G., Lombardi Y., Basic-Jukic N., Aubert O., Dubourg L., Masson I., Mariat C. (2023). Race-free estimated glomerular filtration rate equation in kidney transplant recipients: Development and validation study. BMJ.

[13] Levey A.S., Stevens L.A., Schmid C.H., Zhang Y.L., Castro A.F., Feldman H.I., Kusek J.W., Eggers P., Van Lente F., Greene T. (2009). A new equation to estimate glomerular filtration rate. Ann. Intern. Med..

[14] Gansevoort R.T., Anders H.J., Cozzolino M., Fliser D., Fouque D., Ortiz A., Soler M.J., Wanner C. (2023). What should European nephrology do with the new CKD-EPI equation?. Nephrol. Dial. Transplant..

[15] Delanaye P., Schaeffner E., Cozzolino M., Langlois M., Plebani M., Ozben T., Cavalier E. (2023). The new, race-free, Chronic Kidney Disease Epidemiology Consortium (CKD-EPI) equation to estimate glomerular filtration rate: Is it applicable in Europe? A position statement by the European Federation of Clinical Chemistry and Laboratory Medicine (EFLM). Clin. Chem. Lab. Med..

[16] Cohen J. (1960). A coefficient of agreement for nominal scales. Educ. Psychol. Meas..

[17] Lovrenčić M.V., Biljak V.R., Blaslov K., Božičević S., Duvnjak L.S. (2017). Impact of creatinine methodology on glomerular filtration rate estimation in diabetes. World J. Diabetes.

[18] Syme N.R., Stevens K., Stirling C., McMillan D.C., Talwar D. (2020). Clinical and analytical impact of moving from Jaffe to enzymatic serum creatinine methodology. J. Appl. Lab. Med..

[19] Cheuiche A.V., Soares A.A., Camargo E.G., Weinert L.S., Camargo J.L., Silveiro S.P. (2013). Comparison between IDMS-traceable Jaffe and enzymatic creatinine assays for estimation of glomerular filtration rate by the CKD-EPI equation in healthy and diabetic subjects. Clin. Biochem..

[20] Lee S., Lim L., Chang E., Chiu Y., Hwang S., Chen H. (2019). Effect of differences in serum creatinine estimation methodologies on estimated glomerular filtration rate. Singapore Med. J..

[21] Drion I., Cobbaert C., Groenier K.H., Weykamp C., Bilo H.J., Wetzels J.F., Kleefstra N. (2012). Clinical evaluation of analytical variations in serum creatinine measurements: Why laboratories should abandon Jaffe techniques. BMC Nephrol..

[22] Gottlieb E.R., Estiverne C., Tolan N.V., Melanson S.E., Mendu M.L. (2023). Estimated GFR with cystatin C and creatinine in clinical practice: A retrospective cohort study. Kidney. Med..

[23] Keddis M.T., Amer H., Voskoboev N., Kremers W.K., Rule A.D., Lieske J.C. (2016). Creatinine-based and cystatin C-based GFR estimating equations and their non-GFR determinants in kidney transplant recipients. Clin. J. Am. Soc. Nephrol..

[24] Kukla A., Issa N., Jackson S., Spong R., Foster M.C., Matas A.J., Mauer M.S., Eckfeldt J.H., Ibrahim H.N. (2014). Cystatin C enhances glomerular filtration rate estimating equations in kidney transplant recipients. Am. J. Nephrol..

[25] Delanaye P., Masson I., Maillard N., Pottel H., Mariat C. (2022). The New 2021 CKD-EPI equation without race in a European cohort of renal transplanted patients. Transplantation.

[26] Ku E., Amaral S., McCulloch C.E., Adey D.B., Li L., Johansen K.L. (2022). Comparison of 2021 CKD-EPI equations for estimating racial differences in preemptive waitlisting for kidney transplantation. Clin. J. Am. Soc. Nephrol..

[27] Borrego Utiel F.J., Ramírez Navarro A.M., Esteban de la Rosa R., Bravo Soto J.A. (2020). Comparison of MDRD and the old CKD-EPI equations with the new CKD-EPI equations in kidney transplant patients when glomerular filtration rate is measured with 51Cr-EDTA. Nefrología.

[28] Hundemer G.L., White C.A., Norman P.A., Knoll G.A., Tangri N., Sood M.M., Hiremath S., Burns K.D., McCudden C., Akbari A. (2022). Performance of the 2021 race-free CKD-EPI creatinine- and cystatin C-based estimated GFR equations among kidney transplant recipients. Am. J. Kidney Dis..

[29] Shlipak M.G., Matsushita K., Ärnlöv J., Inker L.A., Katz R., Polkinghorne K.R., Rothenbacher D., Sarnak M.J., Astor B.C., Coresh J. (2013). Cystatin C versus creatinine in determining risk based on kidney function. N. Engl. J. Med..

[30] Levey A.S., Titan S.M., Powe N.R., Coresh J., Inker L.A. (2020). Kidney disease, race, and GFR estimation. Clin. J. Am. Soc. Nephrol..

