# Supplementary material for: The Patient and Treatment Characteristics of Kidney Transplant Recipients with a Clinically Relevant Jaffe/Enzymatic Serum Creatinine Difference
Source: J Clin Med. 2025 Feb 28;14(5):1668. doi: 10.3390/jcm14051668 (PMC11900629; doi:10.3390/jcm14051668)
Supplement: Supplementary file 1 [file jcm-14-01668-s001.zip › jcm-3387429-supplementary.pdf]

Supplement

Supplement Table S1: Proportion of immunosuppressive therapy of adult outpatient kidney transplant recipients at University Hospital Essen, Essen, Germany, 2020-2023

	N [SCr difference ≥ 0.3 mg/dl]		N [all]	% [all] 95% CI
**Immunosuppressive therapy**	**Men**	**Women**		
Tacrolimus, MMF, prednisone	69	8	7,581	1.0 (0.81-1.30)
Tacrolimus, everolimus, prednisone	6	2	827	1.0 (0.49-1.9)
Tacrolimus, belatacept, prednisone	4	1	697	0.7 (0.31-1.70)
Ciclosporin, prednisone	4	1	247	2.0 (0.87-4.60)
Tacrolimus, prednisone	0	2	2,626	0.1 (0.021-0.028)
Everolimus, belatacept, prednisone	0	0	103	0.0

Abbreviations: SCr serum creatinine, MMF mycophenolate mofetil. N reflects the number of measurements.
